# Facile Synthesis of Porous ZnCo_2_O_4_ Nanosheets and the Superior Electrochemical Properties for Sodium Ion Batteries

**DOI:** 10.3390/nano8060377

**Published:** 2018-05-28

**Authors:** Xi Cao, Yang Yang, Aijun Li

**Affiliations:** 1 College of Chemistry and Molecular Engineering, Peking University, Beijing 100871, China; 2 School of Earth and Space Science, Peking University, Beijing 100871, China; sesyxr94@163.com (Y.Y.); liaijun@pku.edu.cn (A.L.); 3 Materials Science & Engineering, Columbia University, New York, NY 10027, USA

**Keywords:** ZnCo_2_O_4_, nanosheet, sodium ion batteries, hydrothermal, anode

## Abstract

ZnCo_2_O_4_ nanosheets with large surface area and mesoporous structure were synthesized using a facile hydrothermal method followed with a calcination process. When applied as the anode material in sodium ion batteries, the ZnCo_2_O_4_ nanosheets demonstrated a high initial charge capacity of 415.1 mAh/g at the current density of 100 mA/g. Even though the reversible capacity decreased in the first 20 cycles, it stayed relatively stable afterwards and retained 330 mAh/g after 100 cycles. This result was superior to those of many reported works of ZnO- and Co_3_O_4_-based anodes for sodium ion batteries, which might be due to the synergistic effect of both Zn and Co, and the refined porous nanosheet-like structure which facilitates electrochemical reactions by providing more reaction sites and ensures cycling stability by providing more space to accommodate the structural strains during cycles.

## 1. Introduction

With the rapid development of portable electronic devices and electric vehicles, different kinds of energy storage devices have been invented. Among them, due to their excellent performance, lithium ion batteries have been extensively studied. However, the safety issues and the limited resources of lithium seem to be the fatal weaknesses for their large-scale application [1,2,3]. Meanwhile, sodium ion batteries are considered to be a potential alternative choice due to abundant sodium resources and their low cost [4,5,6]. In order to achieve higher energy density and longer cycle life, many researchers are working on material innovations; for example, high-specific-energy, high-voltage cathode materials have experienced a blowout in development. Great efforts should also be made to explore new high-capacity materials except for carbonaceous materials with low capacity [5].

As one of the promising anode materials for lithium ion batteries, multicomponent transition metal oxides have received significant research interest due to their distinctive features like high capacity and long cyclability [7,8,9]. Among them, ZnCo_2_O_4_ combines the better anodic performance of cobalt-based oxides and the lower cost/toxicity of Zn. What is more, compared with other ACo_2_O_4_ (where A = Ni, Fe, Cu, and Mg), ZnCo_2_O_4_ has higher theoretical specific capacity as not only can Zn–O and Co–O host lithium ions through conversion reaction, but the alloy of Zn–Li also forms during electrochemical reactions [10,11,12,13,14,15,16]. Based on these considerations, many ZnCo_2_O_4_-based materials with different nanostructures have been synthesized and studied as the anode materials for lithium ion batteries, for example, ZnCo_2_O_4_ hollow nanobarrels [17], ZnCo_2_O_4_ nanoclusters [18], ultrathin ZnCo_2_O_4_ nanosheets [14], and so on. By constructing these different structures, the volume change during cycling can be greatly restrained; the pathway for electron and Li^+^ diffusion can be decreased; and, thus, both rate and cycling performance can be greatly enhanced compared with the bulk materials [11,13,15,16,18,19,20,21].

Based on the great progress of ZnCo_2_O_4_-based anodes in lithium ion batteries, we can also expect superior sodium storing capability of ZnCo_2_O_4_ via similar storage mechanisms to lithium. However, to the best of our knowledge, there are seldom reports concerning the application of ZnCo_2_O_4_ in sodium ion batteries, except for the initial work accomplished by Ru using three-dimensional porous nickel foam as the conductive substrate, with rose-like ZnCo_2_O_4_ grown on it [22]. Although nickel foam is commonly used as the conductive substrate for preparing electrode materials, the heavy self-weight and low loading mass remain the main obstacles for their real application.

In this work, porous ZnCo_2_O_4_ nanosheets were prepared through a simple two-step method, including a facile solvothermal process followed with annealing at 300 °C for 2 h. Compared with other kinds of structures, two-dimensional nanosheets with large surface area exposed to the electrolyte facilitate electron and Na^+^ transport through the material and provide more sites ready for reactions [11,14,22,23,24,25]. As a result, the ZnCo_2_O_4_ nanosheets prepared in this work exhibited a high specific surface area and delivered a high specific capacity, good rate capability, and excellent cycling performance for sodium storage (with a high reversible capacity of 330 mAh/g at 100 mA/g after 100 cycles).

## 2. Experiment Section

### 2.1. Material Synthesis

ZnCo_2_O_4_ nanosheets were prepared through a simple two-step method: Firstly, hexahydrate zinc nitrate, hexahydrate cobalt nitrate, and urea in amounts of 0.2 mmol, 0.4 mmol, and 1 mmol, respectively, were added into a 25 mL Teflon-lined stainless steel autoclave containing 20 mL ethylene glycol and water mixed solution (*v*/*v*, 1:1). The solution was rigorously stirred for 30 min before being put into an oven and reacted at 140 °C for 3 h. Secondly, the ZnCo_2_O_4_ precursor obtained from the hydrothermal reactions was sintered in air at 300 °C for 2 h to get the final product.

### 2.2. Structural Analyses

X-ray diffraction (XRD, D8 Bruker, Billerica, MA, USA) was used to determine the crystal structure of the obtained material. Field Emission Scanning electron microscopy (FESEM, Hitachi S4800, Tokyo, Japan) and transmission electron microscopy (TEM, FEI Tecnai F20, Hillsboro, OR, USA) were used to study the details of the nanostructure. SEM energy-dispersive spectroscopy (EDS) mapping was applied to characterize the element distribution. The specific surface area was measured using the Quantachrome NOVA 4200e system (Boynton Beach, FL, USA). Multipoint Brunauer–Emmett–Teller (BET) and Barrett–Joyner–Halenda (BJH) desorption analyses were performed to obtain the specific surface area and pore size distribution, respectively. The oxidation states of the elements in the ZnCo_2_O_4_ nanosheets were investigated by the means of X-ray photoelectron spectroscopy (XPS) analysis, using the Surface Science Instruments S-probe spectrometer. In order to make the final results credible, three spots were tested on each sample.

### 2.3. Electrochemical Measurements

The ZnCo_2_O_4_ electrode was prepared by mechanical stirring with conductive carbon (Super P) and PVDF (Sigma-Aldrich) at the mass ratio of 8:1:1, with *N*-methyl-2-pyrrolidone (NMP, Alfa Aesar) as the solvent, for several hours. Afterwards, the slurry was spread onto Cu foil and dried in a vacuum oven at 80 °C overnight, with a mass loading of about 1.0 mg/cm^2^. Coin cells were assembled in an argon-filled glovebox (Innovative Technology, IL-2GB), with pure sodium foil as the counter electrode; glass fiber (Whatman GF/A) as the separator; and 1 M NaClO_4_ (Sigma-Aldrich) in a volume ratio 1:1 mixture of propylene carbonate/ethylene carbonate with 5% fluoro-ethylene-carbonate(Sigma-Aldrich) additive as the electrolyte.

The sodium storage capability was studied through galvanostatic discharge and charge measurements using a LAND CT2001A (Wuhan, China). A CHI 605C electrochemical station was used to record the first 3 cyclic voltammetry curves at a scan rate of 0.1 mV/s and impedance with amplitude 5.0 mV in the frequency range from 0.01 Hz to 100 kHz.

## 3. Results and Discussion

**Figure 1:**
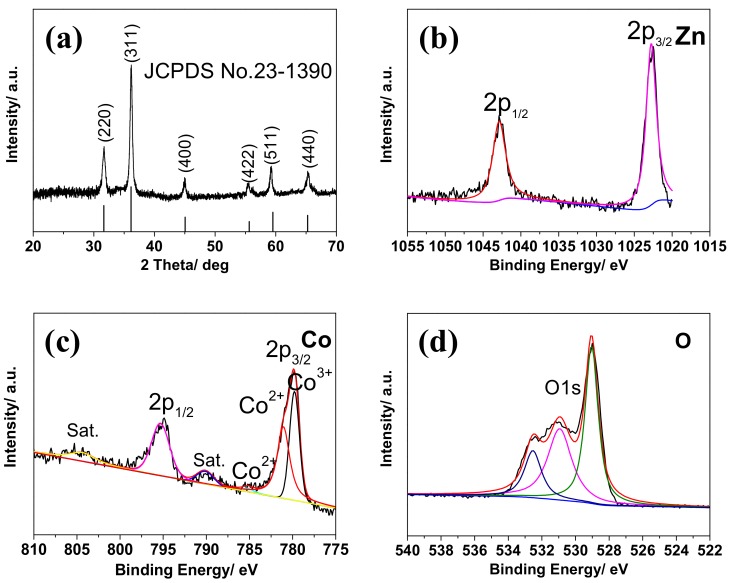
(**a**) XRD patterns of the ZnCo_2_O_4_ nanosheets; (**b**–**d**) XPS results of the ZnCo_2_O_4_ nanosheets.

The XRD pattern of the ZnCo_2_O_4_ nanosheets is shown in Figure 1a. The diffraction peaks were well indexed to the spinel ZnCo_2_O_4_ (JCPDS Card No. 23-1390). No other peaks indexed to impurities could be found from the pattern, indicating high crystallization and purity.

The XPS results of Zn, Co, and O elements in the ZnCo_2_O_4_ nanosheets are shown in Figure 1b–d. Overlapping signals are analyzed using Gaussian-Lorentzian curve after removing the background. In the case of Zn, obviously, two peaks at 1020.58 and 1043.58 eV corresponding to Zn 2p3/2 and Zn 2p1/2 can be seen, which can be ascribed to the oxidation state of Zn^2+^ (Figure 1b). For Co, the two peaks at 779.68 eV and 795.31 eV can be ascribed to Co 2p3/2 and Co 2p1/2 (Figure 1c). The satellite peak observed at the binding energy at 790.1 eV is the characteristic feature of the presence of cobalt in the 3+ oxidation state [26]. There are two weak satellite peaks at 790.1 eV and 804.4 eV, and the energy gap between the main peak and the satellite peaks is around 9–10 eV, which are also a fingerprint for recognition of the Co^3+^ species [18,21]. However, there are still small peaks at around 785.18 eV and 781.0 eV, which suggest the existence of Co^2+^. Figure 1d shows the high-resolution XPS spectra of O, in which the main peaks at 529.5 eV and 531.0 eV can be related to the metal–oxygen bonds, while the several peaks at around 532.7 eV could be interpreted as other contaminants like adsorbed water, chemisorbed oxygen, defects, and so on. All the XPS results analyses above are in agreement with other previously reported works [11,21,22].

**Figure 2:**
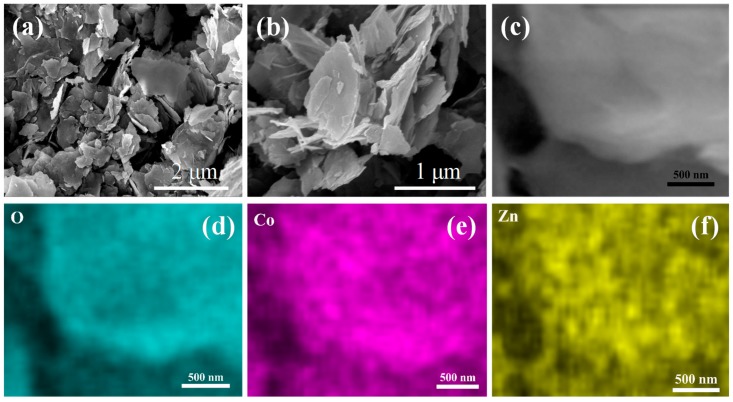
SEM images of the ZnCo_2_O_4_ nanosheets (**a**,**b**) and the EDS mapping images of the ZnCo_2_O_4_ nanosheets (**c**–**f**).

**Figure 3:**
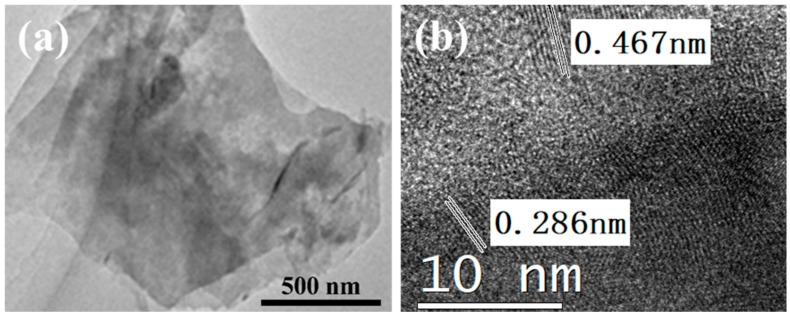
TEM (**a**) and HRTEM images (**b**) of the ZnCo_2_O_4_ nanosheets.

The detailed morphology of the ZnCo_2_O_4_ nanosheets was conducted by scanning electron microscopy (SEM), as shown in Figure 2. Obviously, the ZnCo_2_O_4_ nanosheets showed a two-dimensional morphology with a size from hundreds of nanometers to about 1–2 micrometers. According to a previous report, during the synthesis process, Zn^2+^ and Co^3+^ cations were first interlinked with ethylene glycol molecules to form nanoscale clusters under stirring; then, these nanoclusters began to nucleate and form nanosheets [27]. By controlling the reaction in a short time, the nanosheets would tend to remain [28]. As we can see from the EDS mapping results of the ZnCo_2_O_4_ nanosheets, the three elements O, Zn, and Co were distributed homogeneously on the nanosheets, indicating the high crystallinity of the prepared nanosheets. The structural properties could also be obtained from the TEM and High-Resolution TEM (HRTEM) results, shown in Figure 3. The ZnCo_2_O_4_ nanosheets were transparent under TEM with a size of about 1.5 μm, suggesting their ultra-thin characteristic. It can be observed from Figure 3b that the d spacings of the lattice fringes are 0.286 nm and 0.467 nm, corresponding to the (111) and (220) planes in the XRD pattern.

**Figure 4:**
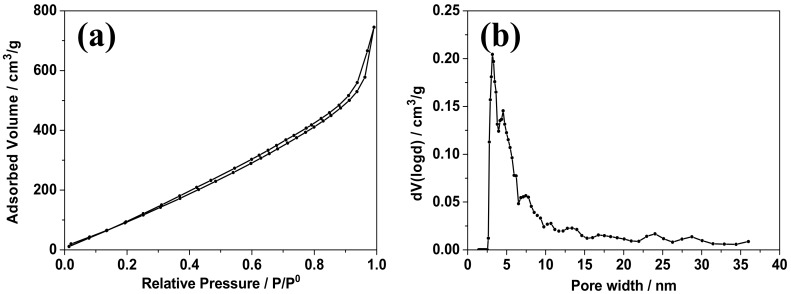
Nitrogen adsorption–desorption isotherms (**a**) and the corresponding Barrett–Joyner–Halenda (BJH) desorption pore size distribution (**b**) of the ZnCo_2_O_4_ nanosheets.

The nitrogen adsorption–desorption isotherms were obtained to determine the specific surface area and the pore size distribution of the ZnCo_2_O_4_ nanosheets, as shown in Figure 4. According to the IUPAC classification, the isotherms demonstrated the H1 type, suggesting the existence of a porous structure [14]. The pore size distribution analyzed using the Barrett–Joyner–Halenda (BJH) model is shown in Figure 4b, and demonstrates a wide distribution of mesopores from 2.5 nm to about 35 nm, but mainly concentrated at about 2.5 to 10 nm; this suggests a mesoporous structure of the nanosheets. The wide pore size distribution above 10 nm may be mainly due to the restacking of the nanosheets. The ZnCo_2_O_4_ nanosheets demonstrated a large specific surface area of about 156 m^2^/g. The large surface area and porous structure may provide more sites for electrochemical reactions and more space to accommodate structure evolution during the cycles; according to previous conclusions [11,14], this may facilitate rate and cycling performance.

**Figure 5:**
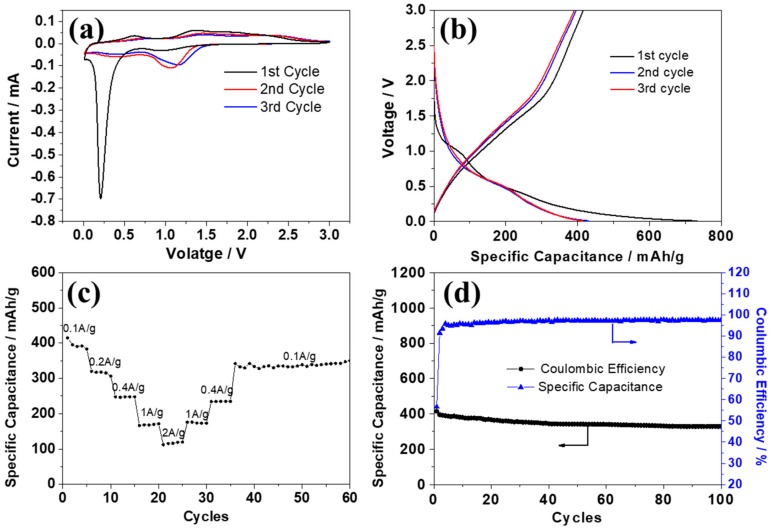
CV curves (**a**) and the first three discharge and charge curves (**b**) of the ZnCo_2_O_4_ nanosheets; Rate (**c**) and cycle performance (**d**) of the ZnCo_2_O_4_ nanosheets at the current density of 100 mA∙g^−1^.

Cyclic voltammogram (CV) curves were first obtained in order to study the electrochemical behavior of ZnCo_2_O_4_ nanosheets as the anode for sodium ion batteries. The first three cycles at the scan rate of 0.1 mV/s in the range of 0 to 3.0 V are displayed in Figure 5a. It can be observed that the first CV curves are obviously different from the subsequent cycles. A slight slope at about 1.10 V may be due to the decomposition of the electrolyte and the irreversible formation of the solid-electrolyte interphase film [29,30]. The sharp peak below 0.5 V could be attributed to the initial reduction of ZnCo_2_O_4_ to Zn and Co, along with the formation of Na_2_O spontaneously. This reaction might be irreversible to some extent; as reported for the ZnO and Co_3_O_4_, a similar peak also existed in the first cycle but did not repeat in the same position in the following cycles [29,30]. As concluded from the literature on ZnCo_2_O_4_ in lithium ion batteries [15,16,18,31], the small cathodic peak at around 0.12 V might be the reduction of Zn to the Na–Zn alloy, even though just a small amount of Zn may participate in this reaction. The anodic peaks at 0.7 V and 1.3 V can be ascribed to the dealloying of Na–Zn to Zn and the oxidation of Zn and Co. After the first cycle, the reduction peak moved to about 1.25 V, which might be ascribed to the polarization effect. The CV curves remained similar from the second cycle, suggesting good structural stability and cycling performance in the sodium ion batteries.

The electrochemical performance of ZnCo_2_O_4_ nanosheets in the sodium ion batteries was further studied via charge and discharge tests at different current densities. Figure 5b demonstrates the first three cycles at the current density of 100 mA/g; the obvious plateau at around 1.1 V and below 0.5 V on the first discharge curve is consistent with the first CV curves, and may refer to the irreversible formation of the SEI film and the partially reversible conversion of ZnCo_2_O_4_ to Zn and Co. The initial discharge and charge capacities reached 733.2 mAh/g and 415.1 mAh/g, respectively, with a coulombic efficiency of about 55.6%. The coulombic efficiency rose to 91.8% and 94.4% in the 2nd and 3rd cycles, even though capacity loss still existed due to volume expansion and structural pulverization. The ZnCo_2_O_4_ nanosheets also demonstrated a moderate rate performance (Figure 5c): when the current densities reached 400 mA/g, 1000 mA/g, and 2000 mA/g, the reversible capacity reached 247.7 mAh/g, 169.6 mAh/g, and 118 mAh/g, respectively. Even though the reversible capacity decreased in the first several cycles, it remained relatively stable even at high rates. When the current density decreased, the reversible capacity gradually increased, and remained 347.3 mAh/g at the current density of 100 mA/g.

The cycle performance was also evaluated in order to study the long-time reaction stability at the current density of 100 mA/g. As demonstrated in Figure 5d, the reversible capacity decreased over the first 20 cycles, from 416 mAh/g to 366.9 mAh/g, but remained relatively stable afterwards, and remained at 330 mAh/g even after 100 cycles. This result was superior to those of many reported ZnO- and Co_3_O_4_-based anodes in terms of cycle stability at a relatively higher current density for sodium ion batteries [29,30,32,33,34], as listed in Table 1. The superior cycling stability and large capacity may due to the synergistic effect of both Zn and Co, and the porous nanosheet-like structure. As illustrated before, the large surface area and porous structure may facilitate electrochemical reactions by providing more reaction sites and, at the same time, ensure cycling stability by providing more space to accommodate structural strain during cycles.

**Figure 6:**
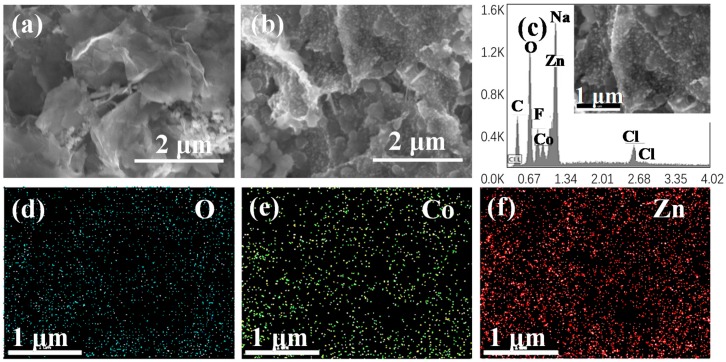
SEM image of the prepared electrode before (**a**) and after cycling (**b**); The EDS mapping results (**c**–**f**) of the electrode after 100 cycles in sodium ion batteries.

The structure and morphology changes of the ZnCo_2_O_4_ nanosheet electrodes were studied via SEM after 100 cycles, coupled with the EDS mapping of O, Co, and Zn. Compared with the electrode before electrochemical reactions in Figure 6a, parts of the ZnCo_2_O_4_ nanosheets retained the sheet-like structure even when parts of them cracked into small particles after cycling due to the volume expansion (Figure 6b), which indicated the relative stability of ZnCo_2_O_4_ nanosheets during the charge/discharge process. The EDS mapping results still demonstrated the homogenously distributed elements of Zn, Co, and O, as shown in Figure 6c–f, with impurities (Na, Cl, and F) originating from the decomposition of the electrolyte (Figure 6c).

## 4. Conclusions

In this work, we prepared porous ZnCo_2_O_4_ nanosheets through a simple two-step method, and studied the sodium storage performance for the first time. The two-dimensional nanosheets, accompanied with the large specific surface area and porous structure, ensured that more surface was exposed to the electrolyte, which facilitated electron and Na^+^ transport through the material, and enabled distinguished electrochemical performance including high reversible capacity, good rate capability, and cycling stability. For example, when the current densities reached 400 mA/g, 1000 mA/g, and 2000 mA/g, the reversible capacity reached 247.7 mAh/g, 169.6 mAh/g, and 118 mAh/g, respectively. The electrode also maintains a high reversible capacity of 330 mAh/g at 100 mA/g after 100 cycles, which might make it an attractive anode material for sodium ion batteries.

## Figures and Tables

**Table 1:** Electrochemical properties comparison of the ZnCo_2_O_4_ nanosheets with ZnO and Co_3_O_4_ as the anode in sodium ion batteries.

Sample	Current Density (mA/g)	Cycles	Specific Capacitance (mAh/g)	Reference
ZnO/rGO/C	48.9	100	300	[29]
ZnO/rGO	48.9	100	164	[29]
Co_3_O_4_/MCNTs	34.2	15	293	[32]
Bowl-like hollow Co_3_O_4_ microspheres	178	10	290	[33]
Electro-spun Co_3_O_4_	90	30	393	[30]
Cobalt oxide needles	89	50	360	[34]
Cobalt oxide slabs	89	50	220	[34]
Cobalt oxide flakes	89	50	167	[34]
Roselike ZnCo_2_O_4_	100	70	444	[22]
**ZnCo_2_O_4_ nanosheets**	**100**	**100**	**330**	**This work**
